# Identification of the Critical Therapeutic Entity in Secreted Hsp90α That Promotes Wound Healing in Newly Re-Standardized Healthy and Diabetic Pig Models

**DOI:** 10.1371/journal.pone.0113956

**Published:** 2014-12-02

**Authors:** Kathryn O'Brien, Ayesha Bhatia, Fred Tsen, Mei Chen, Alex K. Wong, David T. Woodley, Wei Li

**Affiliations:** 1 The Department of Dermatology and the Norris-USC Comprehensive Cancer Center, the Keck School of Medicine, the University of Southern California, Los Angeles, California, 90033, United States of America; 2 Division of Plastic and Reconstructive Surgery, the University of Southern California, Los Angeles, California, 90033, United States of America; Ohio State University, United States of America

## Abstract

Chronic and non-healing skin wounds represent a significant clinical, economic and social problem worldwide. Currently, there are few effective treatments. Lack of well-defined animal models to investigate wound healing mechanisms and furthermore to identify new and more effective therapeutic agents still remains a major challenge. Pig skin wound healing is close to humans. However, standardized pig wound healing models with demonstrated validity for testing new wound healing candidates are unavailable. Here we report a systematic evaluation and establishment of both acute and diabetic wound healing models in pigs, including wound-creating pattern for drug treatment versus control, measurements of diabetic parameters and the time for detecting delayed wound healing. We find that treatment and control wounds should be on the opposite and corresponding sides of a pig. We demonstrate a strong correlation between duration of diabetic conditions and the length of delay in wound closure. Using these new models, we narrow down the minimum therapeutic entity of secreted Hsp90α to a 27-amino acid peptide, called fragment-8 (F-8). In addition, results of histochemistry and immunohistochemistry analyses reveal more organized epidermis and dermis in Hsp90α-healed wounds than the control. Finally, Hsp90α uses a similar signaling mechanism to promote migration of isolated pig and human keratinocytes and dermal fibroblasts. This is the first report that shows standardized pig models for acute and diabetic wound healing studies and proves its usefulness with both an approved drug and a new therapeutic agent.

## Introduction

Rodents such as rats and mice have been the widely used animals for skin wound healing studies. However, these models are less than ideal because they are loose skinned animals and the way they heal skin wounds is predominantly by the mechanism of wound contraction, which may not translate well to human skin wound healing. Pigs, like human beings, are tight skinned animals and heal skin wounds with a larger component of re-epithelialization (i.e. the lateral migration of keratinocytes across the wound bed) and a smaller component of wound contraction. Moreover, pigs are also effective models for topical medication studies, because multiple groups of replicate wounds can be created in the same pig for studies of comparative agents. In randomized wound healing studies, for instance, there is a high concordance of the results between pigs and humans [1]–[4]. However, after careful analyses of the current literature on pig wound healing models, we were surprised to find that few of those previous studies made efforts to first standardize the critical parameters, such as the relationship between locations of wound and their healing rates, optimal distance between two wounds, measurements of diabetic markers over time, correlation between diabetic conditions and delay in wound closure, just to mention a few, prior to using the animals to carry out wound healing studies. There is a need to re-evaluate these parameters and provide established methods for using pigs as wound healing models [1].

At the forefront of wound healing therapeutics, growth factors are thought to serve as the driving force of wound healing and, therefore, have been the focus for therapeutic development of wound healing agents [5]. After decades of investigations and clinical trials, however, the human recombinant platelet-derived growth factor (PDGF) remains the only FDA-approved growth factor for the topical treatment of human diabetic ulcers. This therapy, becaplermin gel (Regranex), has since been shown by multi-center, double blinded and randomized clinical studies to have a modest efficacy. In addition, it showed a fivefold higher potential of causing cancer in patients. Our recent studies identified three molecular hurdles against conventional growth factors and these hurdles could significantly reduce the effectiveness of PDGF-BB/becaplermin gel. First, PDGF-BB only affects dermal fibroblasts, due to the lack of PDGF receptors in human keratinocytes and human dermal microvascular endothelial cells. Second, PDGF-BB-stimulated cell proliferation and migration are completely blocked by the TGFβ family of cytokines, which are abundant in the wound bed. Third, PDGF-BB's biological effects are significantly compromised under the environment of hyperglycemia, the signature for diabetes of all types [6]–[8]. We argue that conventional growth factors simply cannot fulfill the task of promoting wound closure during the critical early phase of wound healing – re-epithelialization.

The above-mentioned disappointing outcomes with conventional growth factors prompted us to search for alternative molecules that could overcome the three obstacles mentioned previously. These efforts led to the discovery of the secreted form of heat shock protein-90alpha (Hsp90α), which is a novel pro-motility factor, resistant to TGFβ and hyperglycemia and has its receptor expressed in every cell type. The topical application of recombinant Hsp90α promotes wound healing in both healthy and diabetic mice [8], [9]. To further establish the importance of Hsp90α in skin wound healing as a potential therapeutic for humans, in this current study, we first establish and re-standardize both acute and diabetic pig wound healing models, including wound size, surgical pattern, correlation between diabetic conditions and delay in wound healing. We provide evidence for the first time that only prolonged diabetic conditions are associated with a delay in diabetic wound healing. Then, we use these models to identify the minimum essential entity in the secreted form of the heat shock protein-90alpha (Hsp90α). We narrow down the therapeutic entity of secreted Hsp90α to a 27-amino acid peptide, called F-8.

## Results

### Comparisons of skin from four preclinical models

**Figure 1:**
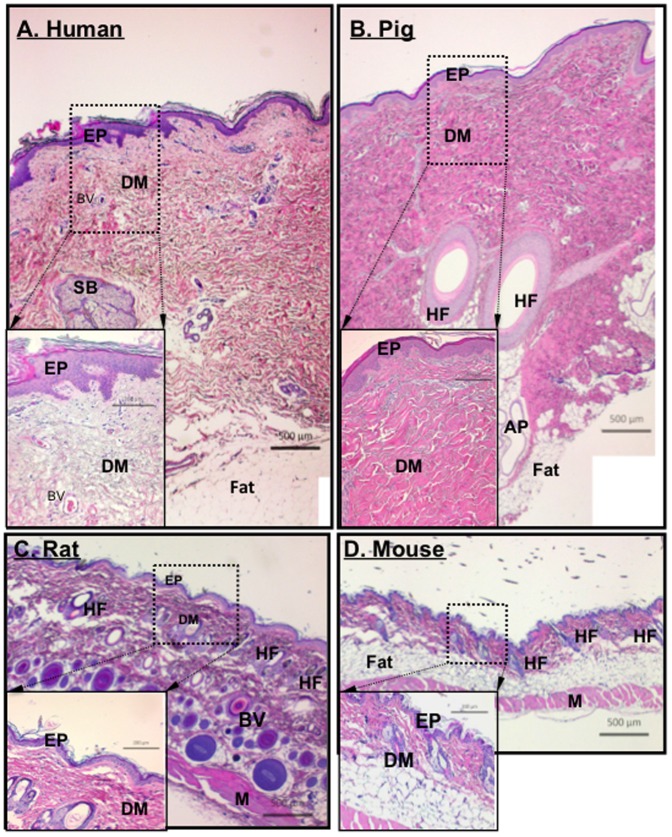
Comparison among human, pig, rat and mouse skin. Paraffin skin sections from various depths of tissue from healthy humans, pigs, rats and mice were simultaneously subjected to H&E staining for structural comparisons. Representative images from each of the four species are shown with the same magnification scale. EP, epidermis; DM, dermis; BV, blood vessel; SB, sebaceous gland, HF, hair follicle, AP, apocrine (sweat) gland; and M, muscle. The measurement bars are as indicated.

We simultaneously evaluated the histology of vertical sections of skin from humans, pigs, rats and mice. The biopsies from rodents and pigs are from similar sites along the spinal region, while the human samples came from different locations due to the variability of the surgical procedures. Nonetheless, similar results were obtained as those presented in Figure 1. Human and pig skin share many similarities in their overall thickness and architecture, including a clear division between the epidermis and the dermis and appearance of appendage (hair follicles, sweat glands, sebaceous glands) distribution (panels A vs. B).

Compared to humans, pigs have less vasculature in their skin and pigs do not have eccrine sweat glands [10] whereas humans have both eccrine and aporcine sweat glands. Rodents have eccrine glands in their foot pads only [11]. The total epidermis of pigs measures 30–140 mm, while human epidermis measures 50–120 mm, rodent epidermis on the other hand measures only 10–45 mm [1], [12], [13]. The turnover time of the epidermal layer is approximately 30 days for pigs, 26–28 days for humans [14], [15] but only 8–10 days for rodents [16], [17]. The combined thickness of the epidermis and dermis in rodent skin is about 10% to 15% that of human skin (Figure 1, panels C and D vs. A and B). The outer most layer of the epidermis is the stratum corneum, here the number of cell layers is similar between pigs and humans; pigs having anywhere between 10–25 layers depending on anatomical location [18], while humans average around 15–25 cell layers [19]. Rodents though typically have only 5 cell layers [20] in their stratum corneum. The turnover rate of the stratum corneum cells is 16 days for pigs [15] and 17 days for humans [21].

Not only is the skin architecture similar between pigs and humans, but so is their wound healing processes. Wound contraction accounts for 90% of wound healing in rodents, while it only accounts for 50% in pigs and 25–50% in humans [22]. The wound contraction in rodents is due to the presence of the subcutaneous panniculus carnosus layer, which is not found in pigs or humans [1]. It is therefore the similarities between the architecture and wound healing processes between humans and pigs that make them an ideal model.

### Relationship between locations and healing rates of wounds in pigs

**Figure 2:**
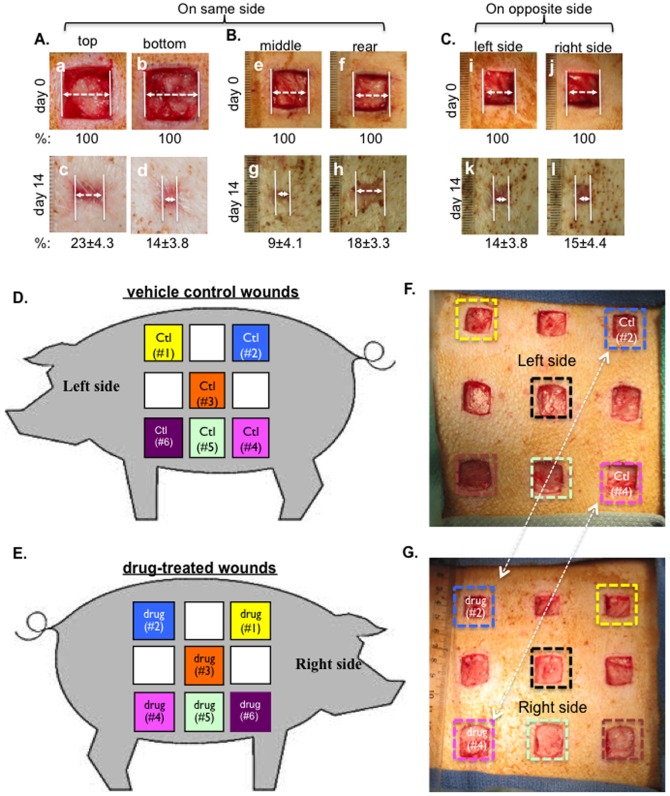
Establishment of the wound pattern for control versus topical drug treatments. (A, B and C) 2.0 cm×2.0 cm full thickness wounds were created on the same side of the pigs (n = 3) with 2.5 cm apart between wounds were compared either between the top and the bottom wounds (A) or between the middle and rear wounds (B) or between wounds made at similar spots, but on the opposite side of the pig (C). Quantitations (% of healing) were made based on triplicate wounds in each pig and shown below each of the images. (D and E) A schematic presentation of nine 2.0 cm×2.0 cm (in normal pigs) or 1.5 cm×1.5 cm (in diabetic pigs) full thickness wounds were created on each side of the pig with 2.5 cm apart between wounds. Comparisons between treatment and control should be made between two wounds at similar spots, but on the opposite side of the pig, as indicated by color squares marked in the same color. (F and G) Based on the above design, real wounds were created on the two sides of pigs. Treatments versus controls are indicated.

Having analyzed previous wound healing studies that used pigs, we came to realize that there has been variability in the utilization of pigs as a wound healing model in different laboratories and there is a need to establish a standard procedure to create and treat pig skin wounds. Specifically, we found that along the left or right side of the torso where the wounds are created, the elasticity, thickness and hair follicle density differ from both top to bottom and from left to right, even within the distance of a few centimeters. The epidermis of the skin becomes more pliable going from the top to the bottom side of the torso. As shown in Figure 2A, wounds that are only a few centimeters from top to bottom (panels a and b) showed significantly different healing rates (panel c vs. panel d). Similarly, as shown in Figure 2B, wounds that are a few centimeters apart from left to right (panels e and f) on the same side of the torso showed different healing rates (panel h vs. panel g). In contrast, wounds created at similar locations, but on the opposite side of the torso, underwent healing at a similar rate (Figure 2C, panel k vs. panel l). Quantitation of the representative experiment is shown as a percentage of the unhealed wound (%) below images. In addition, due to the constant movement of the animal (standing and lying down, running around and scratching against enclosure), exchanges by diffusion could occur between drug-treated and placebo-treated wounds on the same side of the torso. We suggest, as schematically shown in Figure 2D and 2E, wounds should be created on opposite sides of the torso and the wounds should not be randomly matched for a treatment versus its control. Instead, as indicated by the same colored boxes, matching wounds for treatment and control should be at corresponding but opposite sides of the pig's body. Figure 2F and Figure 2G show that real wounds were created on both sides of a pig, in which two matching wounds are marked with the same colored squares for a given treatment and its control.

**Figure 3:**
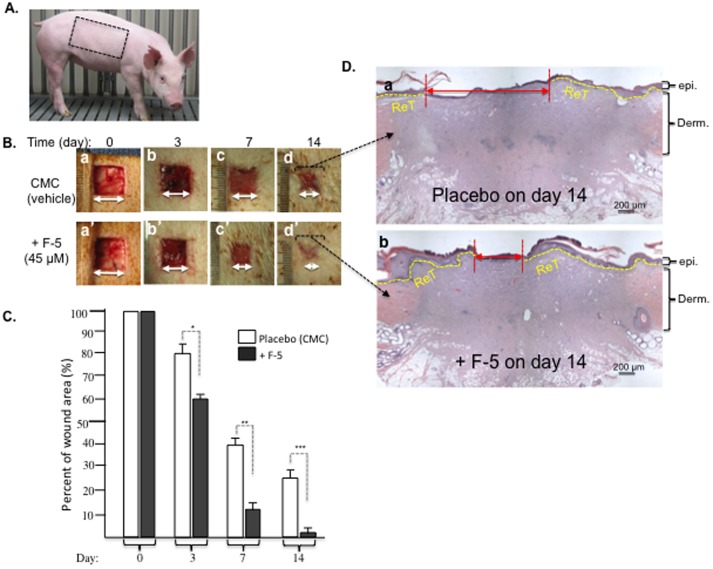
Recombinant F-5 fragment of Hsp90α promoted wound healing in normal pigs. (A) Picture of a typical 25–30 lb and healthy farm pig used in experiments. (B) Wounds (2.0 cm×2.0 cm) in triplicates were topically treated once on day 0 with either CMC gel alone or CMC gel containing recombinant F-5 protein (45 µM). Wounds in triplicate were photographed on the days indicated and analyzed for wound closure rates. (C) Quantitative analyses of the wound closure were presented. (D) Wedge biopsies were made on day 14 wounds, sectioned and stained with H&E. This experiment was repeated four times (a single surgery conducted in four separate pigs) and the results were reproducible. * *p*≤0.05, ** *p*≤0.01 and *** *p*≤0.001, compared with the placebo.

To verify the above design, we topically treated the wounds with recombinant F-5 (amino acids 236 to 350) of human heat shock protein-90alpha (Hsp90α), which we have previously shown to accelerate wound closure in mice [8], [9]. As shown in Figure 3B, topical application of F-5 on day 0 accelerated the wound closure on day 3, 7 and 14 (lower panels, a′ to d′), compared to the control (CMC vehicle) treatment (upper panels, a to d). Quantitation of the data is shown in Figure 3C, which clearly indicates the wound healing-promoting effect of F-5. More encouragingly, H&E staining of the wounds on day 14 revealed that F-5 accelerated the re-epithelialization process, i.e. lateral migration by the keratinocytes at the wound edge, compared to the control (Figure 3D, panel b vs. panel a).

### Shared parameters of human diabetes by STZ-treated pigs

**Figure 4:**
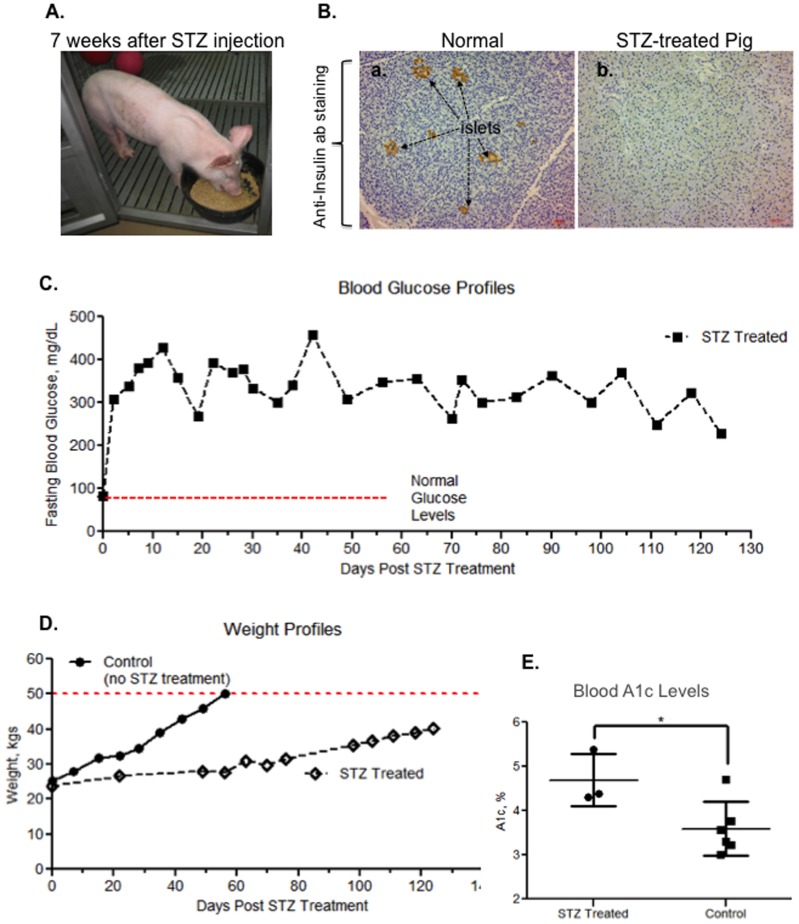
Characteristics of diabetes and delayed wound healing in STZ-treated pigs. (A) A pig, 8 weeks after STZ injection and having undergone the first round of wound healing experiments, was having her breakfast. The pig weighed 36 kgs on that day. (B) Sections of pancreas removed from normal and STZ-treated pigs were subjected to anti-insulin antibody immunohistochemistry staining, the results show complete disappearance of insulin-producing islets in STZ-treated pigs. (C) A typical four-month blood glucose profile of a pig following STZ injection showed hyperglycemia throughout the period of experiments. (D) A typical weight profile of a STZ-treated pig, in comparison to normal pigs. 50 kg is the size limitation (red dotted line) of the facility, when pigs needed to be euthanized. (E) Elevated A1c levels in circulation were detected in three diabetic pigs, in comparison to three controls.

Streptozotocin (STZ) enters β cells through the glucose transporter 2, GLUT2, and causes beta cells to undergo destruction via necrosis, resulting in diabetes in many animal species [23]. Nevertheless, a comprehensive analysis of STZ-induced diabetes in pigs with the defined parameters in diabetic humans was not available in wound healing studies. We examined: insulin-producing islets in the pancreas, blood glucose profiles, body weight profiles and blood A1c (hemoglobin A1c) levels in pigs following STZ injection up to four months. As shown in Figure 4, a representative farm pig appeared normal 7 weeks after intravenous injection with STZ and its completion of the first wound healing study between week 2 and week 4 (see healed wound marks on the right side of its torso). Sections of pancreases removed from either normal or STZ-treated pigs were subjected to anti-insulin antibody immunostaining analysis, which showed complete destruction of the insulin-producing islets from the STZ-treated pig in comparison to a control pig (Figure 4 B, panel b vs. panel a). The blood glucose level rose rapidly to 300 mg/dl within 24 hours following STZ injection and maintained hyperglycemic levels up to four months, whereas normal pigs kept normoglycemia as expected (C, red dotted line). Please note that normal pigs reached the 50 kg weight limit within 60 days and needed to be euthanized in accordance with USDA's current space requirements found in the 8^th^ Edition of the Guide for the Care and Use of Laboratory Animals. STZ-treated pigs gained weight slower than non-diabetic pigs, resembling human diabetic patients (D). The blood A1c level of the STZ-treated pigs increased to an average of 4.6%, in comparison to an average of 3.6% in non-diabetic pigs (E).

**Figure 5:**
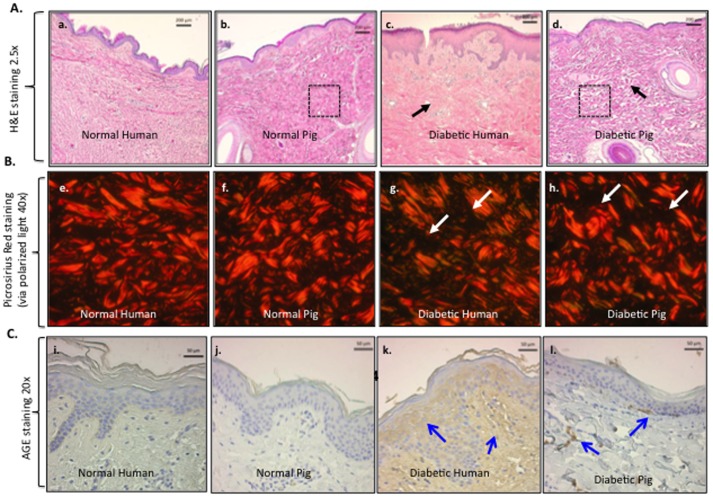
Comparisons between normal and diabetic skin structures in humans and pigs. Comparisons between normal and diabetic skin structures in humans and pigs to investigate if there were similar changes in skin structure between STZ-induced diabetic pigs and humans with diabetes, we obtained skin from diabetic and non-diabetic humans and from normal and STZ-induced diabetic pigs and examined them by H&E histology and immunohistochemistry analyses. (A) H&E staining of normal human skin, normal pig skin, diabetic human skin and diabetic pig skin. The black dotted lines (panels b and d) illustrate the dense ECM structure of normal pig skin compared to the looser ECM structure of the diabetic pig skin. (B) Picrosirius Red staining visualized under polarized light illustrates collagen structure. The white arrows (panels g and h) illustrate the dark areas revealing less density in collagen fibers of the diabetic human and pig tissue compared to their normal counterparts (panels e and f). (C) AGE immunohistochemistry staining reveals less accumulation of Advanced Glycation End Products in the normal human skin compared to the diabetic skin (panel I vs. panel k), see blue arrows. A similar trend is seen in the normal pig skin compared to the diabetic pig skin (panel j vs. l).

Furthermore, H&E histology, Picrosirius Red staining and AGE immunohistochemistry staining analyses showed that diabetic pig skin underwent changes similar to those in diabetic human skin. As shown in Figure 5A, H&E-stained diabetic human skin showed less density in the dermal connective tissue (black arrow, panel c) than normal human skin (panel a). The pixel density of “white space” was measured in 3 equally sized fields of the dermis for each of the different skin samples (no skin appendages were present in the fields measured, see boxes in Figure 5 panels b and d). Density measurements for the normal human sample is 672±37.7 white pixels (wp)/field (f) versus 1450±124.9 wp/f in the diabetic human sample. Similarly, STZ-treated pig skin also exhibited less density of the dermal connective tissues (black arrows) than normal pigs (panel d vs. panel b) although not as strikingly as with the human samples, most likely due to the shorter period of time the pig had been in a hyperglycemic state. The white space density for the normal pig is 639±51.6 wp/f versus 818±38.1 wp/f. These observations were further confirmed by staining collagens with Picrosirius Red and visualized under polarized light. As shown in Figure 5B, the collagen in the dermis of diabetic human (panel g) and diabetic pig (panel h) were disorganized, in comparison with the non-diabetic skin controls (panels e and f). Moreover, both diabetic human and diabetic pig skin showed increased glycation levels. As shown in Figure 5C, AGE staining showed overall increased glycation in diabetic human skin (panel k vs. panel i). Similarly, in diabetic pig skin, a scattered but significant increase in glycation, again likely due to the much shorter period of hyperglycemia, was clearly detected (panel l), in comparison to the control (panel j) These results indicated that STZ-treated pigs exhibited the characteristics of diabetes similar to those in humans.

### Identification of the onset for delayed wound healing in STZ-treated pigs

**Figure 6:**
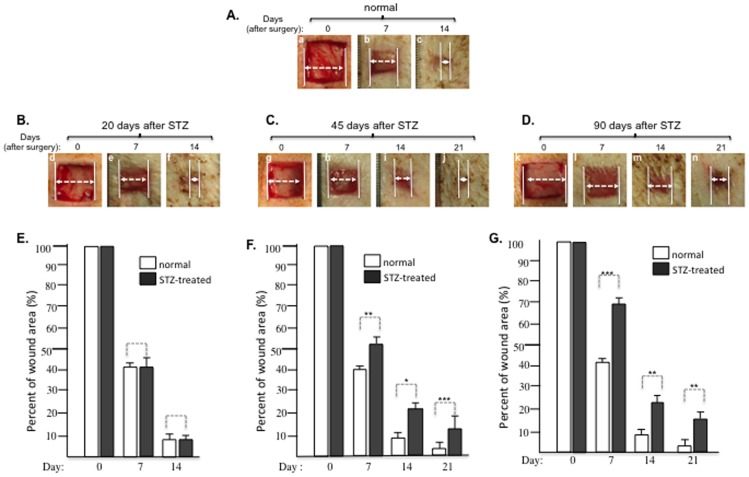
Duration of diabetic conditions correlated with the length of delay in wound healing. The length (days) of delay in wound healing was examined in pigs injected with STZ for 20, 45 and 90 days prior to wound surgery. (A) Images of the 1.5 cm×1.5 cm full thickness wounds made in a representative normal pig; (B) Images of the wound healing in a representative pig 20 days after STZ injection; (C) Images of wounds in a representative pig 45 days after STZ injection; (D) Images of wounds in a representative pig 90 days after STZ injection; (E to G) Quantitative analyses of the wound healing data shown in B, C and D (n≥3) in comparison to wounds shown in A. * *p*≤0.05, ** *p*≤0.01 and *** *p*≤0.001, compared with placebo.

Delayed healing is the clinical signature of diabetic skin wounds in humans. A previous study reported 4 to 6 days of delay in skin wound closure in pigs that were wounded 14–20 days following STZ injection [24]. However, we were unable to detect any significant delay in wound healing in pigs under similar conditions. We postulated that it might need to take a longer period of time for diabetic conditions to cause any significant delay in wound healing. To test this hypothesis, we conducted a series of wound healing experiments in pigs whose diabetic conditions were kept for 20, 45 and 90 days prior to the surgical procedures. As shown in Figure 6A, 1.5 cm×1.5 cm full thickness wounds in control pigs healed around day 14 (panels a, b, c). Similar wounds did not show any significant delay in the pigs 20 days following STZ injection (6B, panels d, e, f), after quantitation (6E). However, we started to detect a significant delay in wound closure in pigs 45 days following STZ injection (Figure 6C, panels g, h, i, j vs. panels a, b, c). The wounds clearly remained open on day 14 (panel i) and closed around day 21 (panel j). Quantitation of the data showed 12–15% delay in wound closure (Figure 6F). More convincingly, in pigs 90 days following STZ injection prior to surgery, the wounds remained open even on day 21 (Figure 6D, panel n). Quantitation showed 18–30% delay in wound healing (Figure 6G). We were unable to continue the experiments beyond 90 days following STZ injection, due to the 50 kg weight limit set by the USDA space requirements for pigs.

### F-5 versus becaplermin (PDGF-BB) gel on promoting wound healing in diabetic pigs

**Figure 7:**
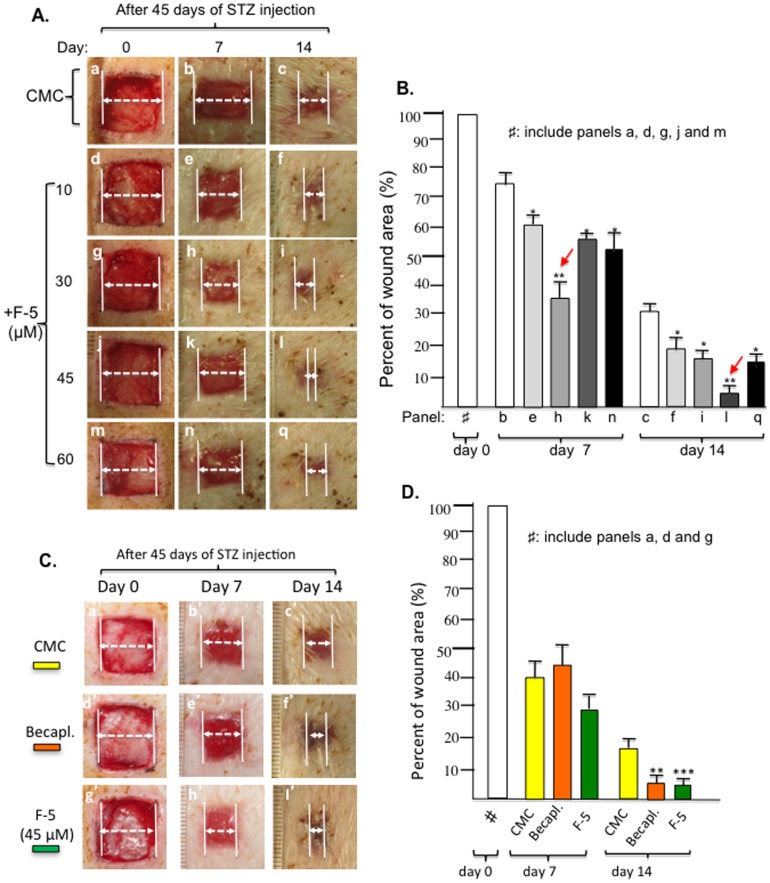
Comparison of F-5 with becaplermin gel in promoting diabetic pig wound healing. (A) Images of 1.5 cm×1.5 cm full thickness wounds in triplicates in pigs 45 days following STZ injection were topically treated with increasing amounts of recombinant F-5 protein once on day 0. Wound closure was measured on day 7 and day 14. (B) Quantitative analysis of the wound closure data revealed an optimal concentration for F-5 between 30 µM to 45 µM. * *p*≤0.05 and ** *p*≤0.01, compared with placebo. (C) Images of similar wounds were topically treated with 45 µM of F-5 protein or becaplermin gel as prescribed in triplicate on day 0. Wound closure was compared on day 7 and day 14. (D) Quantitative analysis of F-5- and becaplermin gel- stimulated wound closure data. * *p*≤0.05, *p*≤0.01 and *** *p*≤0.001, compared with placebo.

Using the above diabetic pig model, we tested whether F-5 is also able to correct delayed wound healing. On wounds in pigs 45 days following STZ administration, as shown in Figure 7A, 10 µM of F-5 showed a modest promotion of wound closure on both day 7 and day 14, in comparison to placebo controls (panels d, e, f vs. panels a, b, c). A greater enhancement was observed with 30 µM of F-5, especially on day 7 (panels h vs. panels b and e). The 45 µM F-5 showed rather a weaker stimulatory effect on day 7, with the strongest effect on day 14, leading to complete closure of the wounds (panel l vs. panels c, f or i). The 60 µM F-5 showed little further improvement on day 7 and a slightly inhibitory effect on day 14, in comparison to 45 µM of F-5 (panels n, q vs. panels k, l). Quantitative analyses of these experiments are shown in Figure 7B. These results indicated a plateau effect for F-5 in diabetic pig wounds between 30 µM to 45 µM.

**Figure 8:**
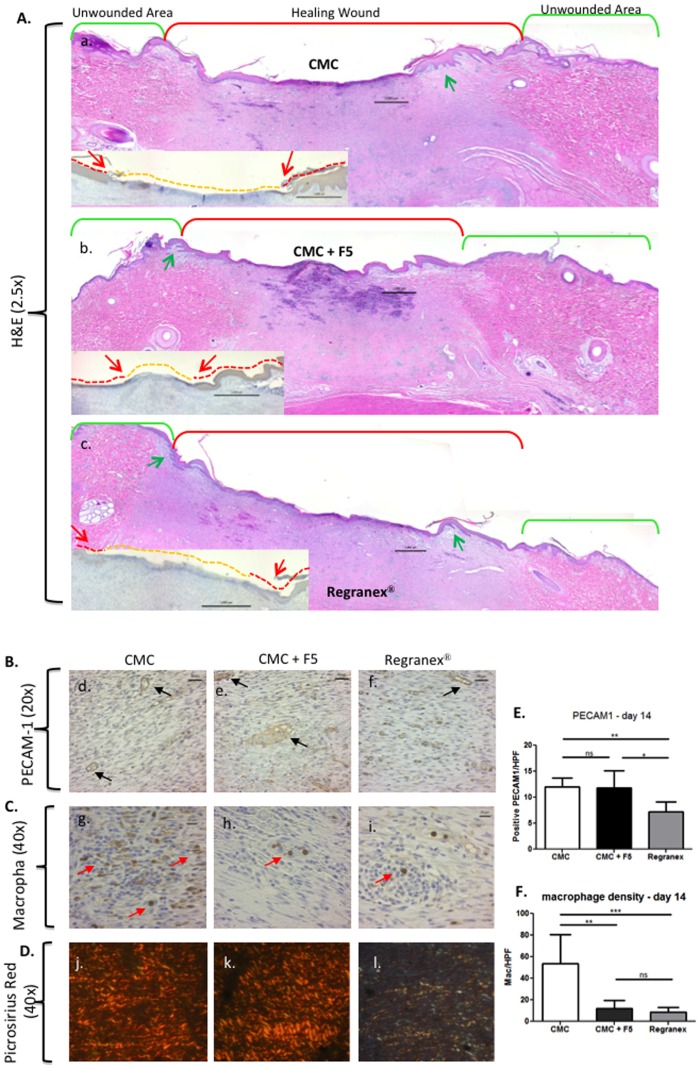
Histological analyses of healed wounds treated with F-5 versus becaplermin. Skin biopsies of CMC, F-5-treated and becaplermin-treated diabetic pig wounds on day 14 were subjected to various histochemistry and immunohistochemistry analyses. (A) H&E staining showed rete ridge production between the epidermis (green arrows) and dermis (panel b vs. panel a and panel c). Insert is pan keratin antibody staining showing the re-epithelialization tongue (red line and red arrows), the orange line shows the unhealed area devoid of epidermis. (B) Anti-PECAM-1 antibody staining indicated more blood vessel formation in the newly healed wound site of both F-5 treated wounds and CMC control compared to the becaplermin treated wounds (panels d and e vs. panel f). (C) Anti-Calprotectin antibody for macrophage staining (red arrows) shows more inflammatory cells are present in the CMC control compared to either the F-5 treated wounds or becaplermin treated wounds (panels h and I vs. panel g). (D) Picrosirius Red staining with polarized light microscopy confirmed better organized dermis in the F-5-treated wounds than the CMC control or becaplermin treatment (pane k vs. panel j and panel l). (E and F) Quanitative data for PECAM-1 positive staining per high powered field (HPF) (E) is given as well as the number of macrophages per HPF (F). * *p*≤0.05, ** *p*≤0.01 and *** *p*≤0.001. The above data represent a consensus from multiple and non-continuous sections of a given skin specimen.

We chose the 45 µM dosage of F-5 and compared its treatment with the becaplermin gel treatment. As shown in Figure 7C, delayed wound healing was observed in CMC alone-treated wounds that remained open on day 14 (panels a′, b′, c′). Treatment with becaplermin gel accelerated the wound closure on day 7 and day 14 (panels d′, e′, f′). In parallel, treatment with F-5 showed a comparable stimulatory effect on day 7 to becaplermin gel (panel h′ vs. panel e′) and a stronger effect on day 14 than becaplermin gel (panel l′ vs. panel f′). Quantitative analysis of these results is shown in Figure 7D. Interestingly, as shown in Figure 8, histochemistry and immunohistochemistry analyses showed that the F-5-treated wounds exhibited enhanced re-epithelialization (insert A, panel b), more organized collagen deposition (B, panel k) than becaplermin gel or the CMC alone treated wounds. Also, both F-5 and CMC control treated wounds had a similar amount of blood vessel formation over becaplermin treated wounds (B panels d and e vs. f). Finally, both F-5 and becaplermin treated wounds showed less inflammation with fewer macrophages present in the wound bed over the CMC alone treated wounds (C panels h and i vs. g).

### 27-amino acid peptide, F-8, determines extracellular Hsp90α function in wound healing

**Figure 9:**
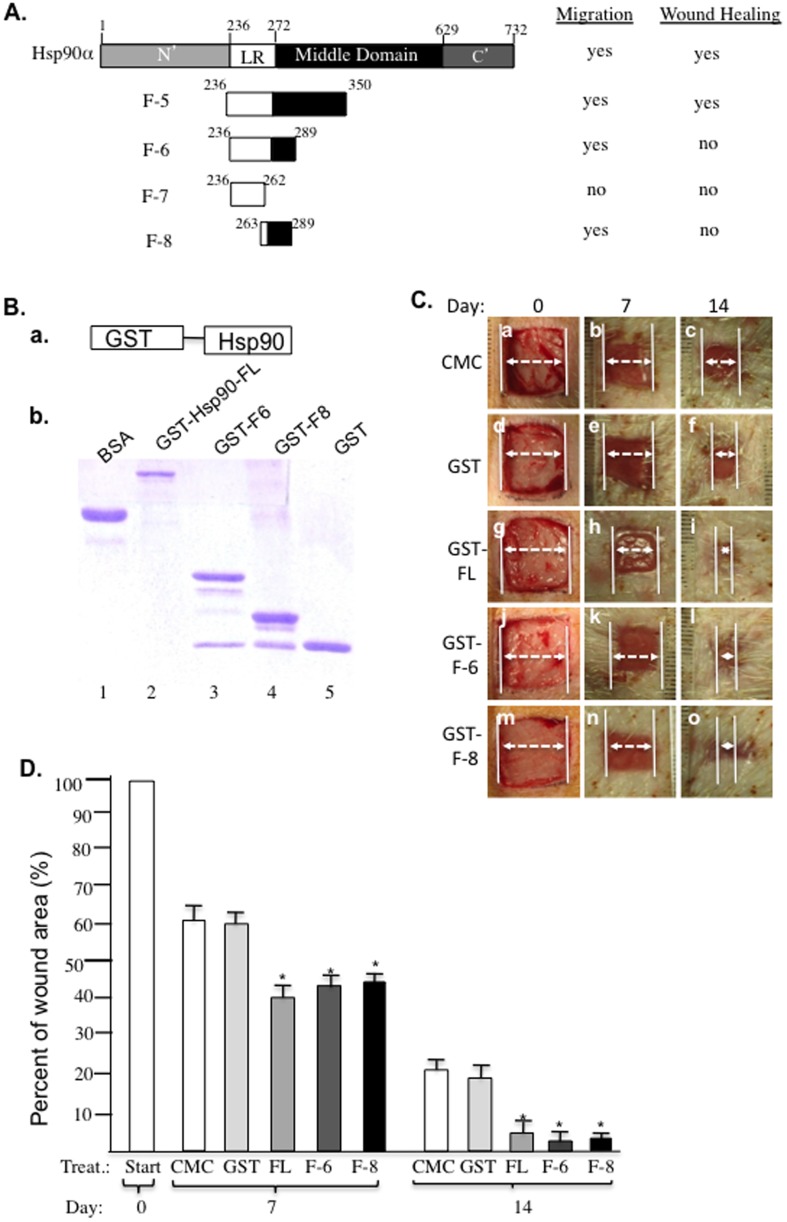
A 27-amino acid peptide, F-8, determines the wound healing effect of Hsp90α. (A) A schematic representation of mutagenesis of Hsp90α down to the minimum peptides of 27 amino acids and the profiles of their pro-motility activities. (B) GST-fusion proteins were generated as shown in a SDS gel stained with Coomassie blue. (C) Effects of the various GST-fusion proteins (300 µg/ml for all) on wound healing in pigs, which followed the procedures as shown in Figure 4. (D) Quantitation of the wound healing data in triplicate. * *p*≤0.05 compared with placebo.

A peptide that reaches its minimum size and still retains its function is preferred for therapeutic development, because of its higher specificity and lower off-target effects, especially when an unrelated carrier protein can be used to correct its possibly compromised stability. This concept prompted us to further identify the minimum size of Hsp90α that still retains the pro-motility activity of Hsp90α in vitro and enhanced wound healing effect in vivo. Deletion mutagenesis of the F-5 fragment, as schematically summarized in Figure 9A, led to the 54-amino acid peptide, called F-6, that retained the pro-motility effect of F-5. When F-6 was further shortened into two 27-amino acid peptides, called F-7 and F-8, we found that only F-8, but not F-7, still retained a majority of the pro-motility activity of F-5. We were unable to further obtain a functional peptide that is shorter than F-8. However, we found that neither F-6 nor F-8 peptide alone was able to promote pig wound healing. To test the possible compromised stability of these peptides in the wound environment, glutathione s-transferase (GST) was linked to F-6 and F-8 as a carrier protein. As shown in Figure 9B, GST-FL (Hsp90α), GST-F-6, GST-F-8 and GST alone were shown in an SDS-PAGE gel with BSA as control. Interestingly, when GST-F-6 and GST-F-8 proteins were topically applied to normal pig wounds, as shown in Figure 9C, both GST-F-6 (panels j, k, l) and GST-F-8 (panels m, n, o) were able to promote wound healing as much as GST-FL Hsp90α did (panels g, h, i), in comparison to the CMC control (panels a, b, c) or GST alone (panels d, e, f). Quantitation of the data is shown in Figure 9D. Thus, we concluded that F-8 is the minimum size of Hsp90α that retains the therapeutic effect on wound healing. Although the GST-F-8 fragment has not been tested in diabetic pigs at this time, based on the efficacy of the F-5 fragment in diabetic pig wounds, similar efficacy is expected of the GST-F-8 fragment.

### Hsp90α promotes pig and human cell migration via LRP-1 receptor

**Figure 10:**
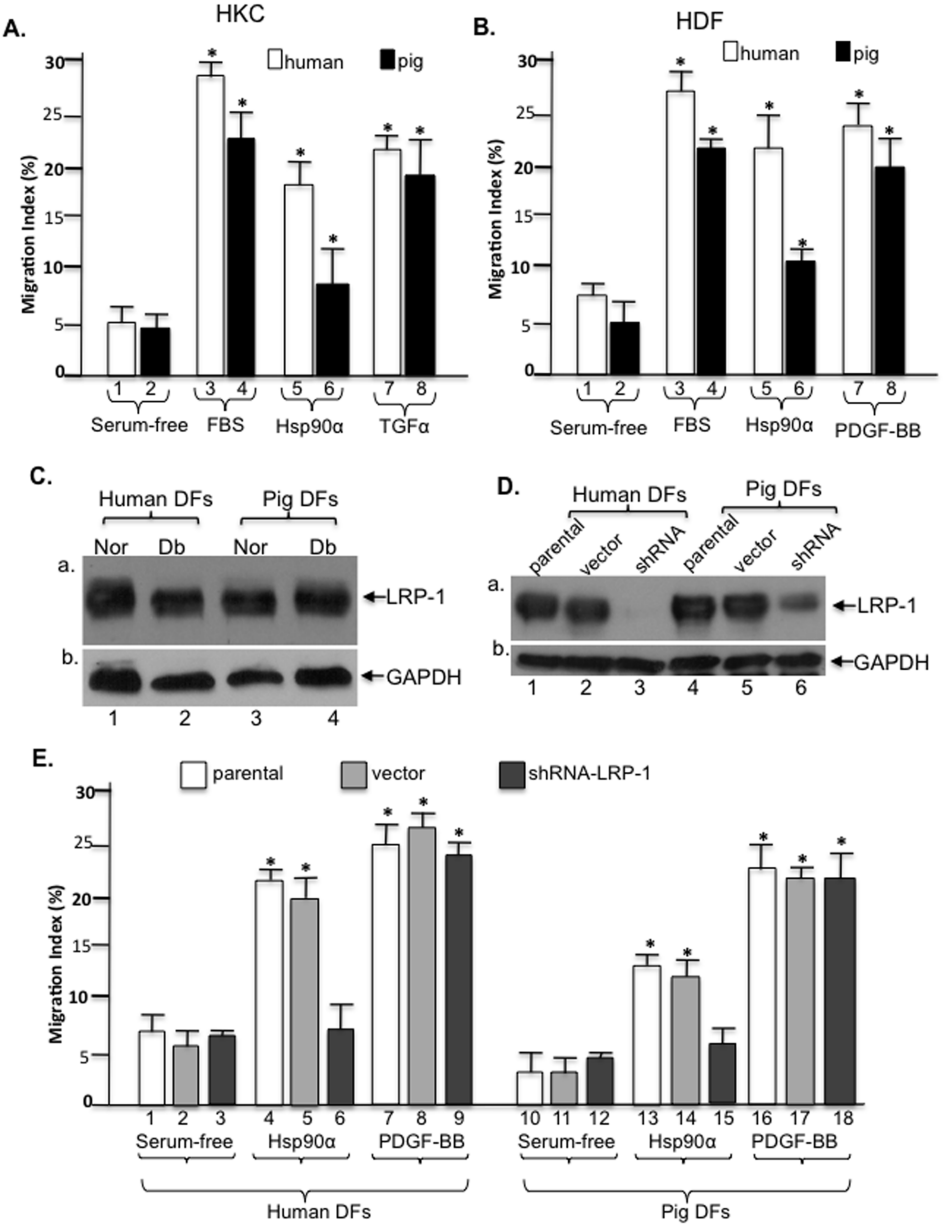
Comparison of F-5 on human versus pig cell migration. Primary human and pig keratinocytes and dermal fibroblasts were isolated. (A) Motility of the serum-starved human and pig keratinocytes on type I collagen in response to FBS (10%), Hsp90α (10 µg/ml) or TGFα (20 ng/ml). (B) Motility of human and pig dermal fibroblasts in response to FBS (10%), Hsp90α (10 µg/ml) or PDGF-BB (15 ng/ml). Quantitative analysis of the above migration assays is presented as Migration Index (%). (C) Lysates of the normal (Nor) and diabetic (Db) human and pig dermal fibroblasts were subjected to anti-LRP-1 antibody immunoblotting. (D) Down-regulation of LRP-1 in both human (lane 3) and pig (lane 6) dermal fibroblasts was confirmed by Western blot with anti-LRP-1 antibody. (E) The cells shown in panel D were subjected to colloidal gold migration assay in response to F-5 or PDGF-BB stimulation. Down-regulation of LRP-1 blocked F-5-stimulated (bars 6 and 15), but not PDGF-BB-stimulated (bars 9 and 18), dermal fibroblast migration. This experiment was repeated four times and reproducible results obtained. * *p*≤0.05.

We then tested whether Hsp90α promotes pig cell migration like it does to human cells in vitro and, more importantly if it uses a similar mechanism. Pig and human keratinocytes and dermal fibroblasts were isolated from surgical specimens and subjected to migration assays. As shown in Figure 10A, FBS and TGFα equally stimulated both pig and human keratinocyte migration (bars 3, 4 and 7, 8 vs. bars 1 and 2). Interestingly, Hsp90α-stimulated human keratinocyte migration appeared to be significantly stronger than Hsp90α-stimulated pig keratinocyte migration (bar 6 vs. bar 5). Similar observations were made for dermal fibroblast migration. As shown in Figure 10B, while FBS and PDGF-BB stimulated pig and human dermal fibroblast migration to a similar degree (bars 3, 4 and 7, 8 vs. bars 1 and 2), Hsp90α stimulated a much stronger migration effect of human dermal fibroblasts than pig dermal fibroblasts (bar 6 vs. bar 5). If one extrapolates these results, in conjunction with the F-5 and PDGF-BB stimulated wound healing (see Figure 7C), it suggests that F-5 may only have shown half of its real efficacy in the pig wounds than it might be able to show in human wounds.

Finally, we studied whether Hsp90α stimulates pig and human cell migration via the same mechanism, i.e. LRP-1 (LDL-receptor-related protein-1). As shown in Figure 10C, human and pig dermal fibroblasts (normal or diabetic) express similar levels of LRP-1 (panel a), the cell surface receptor for Hsp90α [7]. Using lentiviral infection, as shown in Figure 10D, we were able to achieve nearly complete knockdown of LRP-1 expression in human cells (lane 3) and approximately 70% knockdown of LRP-1 expression in pig cells (lanes 6), in comparison to their corresponding human (lanes 1, 2) and pig (lanes 4, 5) controls. When these cells were subjected to the colloidal gold migration assay, as shown in Figure 10E, Hsp90α was no longer able to promote migration of the LRP-1-downregulated human (bar 6 vs. bars 4 and 5) and pig (bar 15 vs. bars 13 and 14) cells. In contrast, down-regulation of LRP-1 did not affect PDGF-BB-stimulated human (bar 9 vs. bars 7 and 8) and pig (bar 18 vs. bars 16 and 17) cell migration. These results clearly indicated that Hsp90α uses a similar signaling mechanism to promote pig and human cell migration.

## Discussion

Wound healing studies in live animals remain the most predictive method to gain insights into the wound healing mechanisms in humans. Among the currently used animal models, it is widely accepted that pigs have skin that is both histologically and functionally closer to humans' and, therefore, have widely been regarded as the “right” model for wound healing studies [1]. Yet, when we started searching for established protocols using pig wound healing models prior to our study, we surprisingly found that there was a complete lack of standardized methods and measurements or treatments and controls. Since 2008, for instance, there have been a handful of wound healing studies using STZ-induced diabetic pigs and yet the reported findings were inconsistent [24]–[30]. Studies from Eriksson's group created full-thickness skin wounds in diabetic pigs 14 days after STZ injection. Based on Hematoxylin and Eosin (H&E) staining, they reported that complete re-epithelialization of 1.5 cm×1.5 cm full thickness wounds occurred on day 12 to 14 for non-diabetic pigs and on day 18 for STZ-treated pigs [24]. Bergmann *et al* compared partial-thickness wounds on normal and diabetic pigs and reported little difference in wound closure rates [28]. Except for these two studies, the majority of other studies only examined STZ-induced diabetic pigs and did not make any comparisons to normal healthy pigs or did not pay particular attention to whether or not wound healing was delayed in those pigs [25]–[27], [29]. Consequently, the results of our initial experiments that followed the procedures in those studies were highly variable and un-reproducible. We concluded that there was a need to first establish the methodology of using pigs as a wound healing model. In this current study, we systematically evaluated all critical parameters and standardized both healthy and diabetic wound healing models in pigs. the new standards include (i) a wound-creating pattern for therapeutic treatment versus the control; (ii) measurements of the physiological parameters of diabetes, (iii) demonstration of effectiveness for a FDA-approved wound healing agent to support the relevance of these models; and (iv) most importantly, identification of a 27-amino acid peptide, called F-8, as the core entity of Hsp90α to promote wound healing.

What is the physiological relevance of Hsp90α secretion to diabetic wound healing? The answer points to the levels of the key cellular responding protein to environmental hypoxia, the hypoxia-inducible factor-1alpha (HIF-1α). We previously reported that the hypoxia-driven secretion of Hsp90α is under direct control of cellular HIF-1α levels [9], [31]. Impaired reaction to hypoxia is known to contribute to impaired wound healing, such as in diabetic ulcers [32]. Lower levels of HIF-1α protein were found in foot ulcer biopsies in patients with diabetes, in which hyperglycemia was shown to reduce the HIF-1α stability and function [33]–[35]. These findings suggest that delayed diabetic wound healing is the result of HIF-1α destabilization and provide strong support for topical treatment of diabetic wounds with recombinant Hsp90α protein to bypass the damaged HIF-1α in human diabetic wounds. While it remains to be tested whether the HIF-1α levels are affected in our diabetic pig model, our study clearly shows that the topical application of Hsp90α proteins greatly accelerated wound closure in these pigs.

It was argued that the available diabetic animal wound healing models only demonstrate a short-term impairment in the wound repair process and, therefore, may not reflect the true nature of chronic wounds in humans that can persist for years. Hence these diabetic wound models are actually models for impaired acute wound healing rather than true chronic wounds [36]. Given the life span of current experimental animals (i.e. only a fraction of humans') and the variability in the biology of human wounds, it is true that there is no perfect animal model for human skin wound healing studies. Our data herein clearly show for the first time that the longer the condition of diabetes is sustained in pigs the more evident a delay in wound healing takes place. This finding is consistent with the clinical observations on diabetic foot ulcers in humans. If we extrapolate the findings from our study, topical application of recombinent Hsp90α proteins would show promising results in future clinical trials.

## Materials and Methods

### Animals

Female Yorkshire pigs (S&S Farms, Ramona, CA) 2 to 3 months in age and weighing 20–25 kgs at arrival were acclimated for at least one week prior to experimental procedures. Six normal pigs were used for non-diabetic control wound studies. An additional six pigs were made diabetic by STZ injection, among which one pig died 5 days after STZ induction of unknown etiology. The other five pigs were maintained in a diabetic state throughout the periods of experiments with fasting blood glucose levels above 200 mg/dl.

### Studies in non-diabetic healthy pigs

During the initial “wound pattern” studies, 2.0 cm×2.0 cm full-thickness wounds were created in healthy pigs exactly following the procedures as described in detail below.

### Induction of hyperglycemia by STZ in pigs

STZ (Enzo Life Science, Farmingdale, NY) was prepared in 0.9% saline (Teknova, Hollister, CA), sterilized by filtration through a 0.22 µm filter and administered at 150 mg/kg of body weight after the pig was sedated. The intravenous injections were carried out over 15–20 minutes. Fasting blood glucose levels were tested twice weekly with “Freestyle Lite” glucose monitor and test strips (Abbott Diabetes Care, Alameda, California). The blood glucose levels were sustained between 250 and 450 mg/dl during the course of the experiments whether it was one month or four months, largely by controlling the daily food intake of the animals. Humulin N insulin (Eli Lilly, Indianapolis, Indiana) was also planned to be given intravenously to the pigs if the glucose levels rose to 600 mg/dl or higher in order to avoid possible ketoacidosis. To test the blood levels of A1c, 1 cc of blood was collected from the ear vein and tested by Antech Diagnostics (Irvine, CA) for the averaged concentration of glycated hemoglobin. A1c serves as a marker for the average blood glucose levels for the previous period of three months.

### Creating the pattern of multiple wounds for control and treatments

A new pattern of wounds for fair comparative studies is recommended as follows. Wounds on one side of the pig were entirely used for control treatments (sterile carboxymethyl cellulose, CMC) and wounds on the other side of the pig used for treatments of the peptides of interest (see the text for details). All surgical procedures took place under sterile conditions in a designated operating room. Animals were pre-medicated with Ketamine/Xylazine 2.2–4.4 mg/kg. Once sedated, animals were intubated and maintained with 1–4% Isoflurane continuous inhalation. Intramuscular injections of Bupronorphene at 0.02–0.05 mg/kg and Carprofen tablets at 2–4 mg/kg were used as post-operative analgesics. Under anesthesia, the pig's sides were shaved and prepared with betadine scrub and solution. Wounds were created on day 14, 20, 45 or 90 following STZ injection. Depending upon design of a given experiment, nine (in three rows) to twelve (in four rows) 1.5 cm×1.5 cm squares were outlined using permanent black marker around a premade template on the pig's torso. This area was washed with ethanol and prepped with sterile drapes. The distance between two wounds is 2.5 cm. Using number 15 scalpel blades the wounds were cut to a full thickness depth; the epidermis, dermis and underlying fat were removed to expose the fascia layer below. The depths of the wounds were measured at approximately 5 mm. We recommend that the maximum number of wounds on each side is 12, making a possible maximum of 24 wounds for each surgery in a pig.

FDA-approved becaplermin gel (Regranex from Smith and Nephew, Andover, MA, or recombinant human PDGF-BB) was used as a positive control for the treatment of diabetic wounds. Recombinant full-length or the F-5 fragment of Hsp90α were mixed in 0.3 gm of 15% sterile CMC. These drug or tested Hsp90α proteins were topically applied on wounds in triplicates in a pig. Wounds were then covered with Opsite clear bandages (Smith and Nephew, Hull, UK), overlaid with a cotton gauze cloth taped to cover the entire wound area. Finally, the entire area that included all the wounds on two sides of the pig is wrapped (360°) in elastic bandages, followed by a final wrap in Elastikon (Johnson & Johnson, New Brunswick, NJ).

### Measurements of Wound Healing

After surgery and various treatments, digital photographs were taken individually of each wound on day 0, 7, 14, and 21 from a fixed distance. Wound healing was analyzed based on measurements of the wound closure and histology/immunohistochemistry. To measure wound closure, the area of an open wound on that day was measured and compared to the area of the wound on day 0 following surgery, using the software AlphaEase FC version 4.1.0 (Alpha Innotech Corporation, Miami, FL), as previously described [8], [9]. The histological analyses were carried out for skin wounds and the pancreas. Wedge biopsies measuring 2 cm×2 cm were taken on day 14, 21 or otherwise specified day for skin wounds. The pancreas was removed on the day when the animal was sacrificed. All tissue samples were fixed in 10% formalin (VWR, Randor, PA), and placed in paraffin blocks for sectioning. Immunohistochemistry studies on skin wounds were conducted with anti-PECAM-1 (1∶100, SC-1506, Santa Cruz Biotechnology, Santa Cruz, CA), anti-AGE (1∶1000, ab23722, Abcam), anti-pan keratin (1∶100, ab8068, Abcam) and anti-calprotectin molecule for macrophage staining (1∶100, mA1-80446, Thermo Fisher Scientific, Rockford IL) antibodies. Sections of the pancreas from normal and diabetic pigs were immunostained with anti-insulin antibody (1∶50, GTX73558, GeneTex), according to previously detailed procedures [8]. Sections were also stained via Picrosirius Red and H&E. PECAM-1 (capillary lumen) density in the wound beds were measured as the average number of PECAM-1 positive lumens from five high powered fields (HPF, 20X) per section. Analysis was performed by a pathologist who was blinded to the treatment groups. Similarly, the number of macrophages in seven high powered fields (HPF, 20X) were averaged [38].

### Isolation of primary skin cells from pigs and humans

Human skin samples from patients obtained with informed consent for elective surgeries were washed with PBS then placed in PBS containing 25 caseinolytic units/ml of dispase (BD Bioscience, San Jose, CA) and incubated overnight at 4°C. The skin samples were washed with PBS and the epidermis was separated from the dermis by a set of sterilized surgical tools. To isolate keratinocytes, the epidermis was placed in 0.25% trypsin-EDTA solution (Gibco, Life Technologies, Grand Island, NY) for 20 minutes at 37°C and the digestion reaction stopped by the addition of soybean trypsin inhibitor. The cell and tissue mixture was poured through a cell strainer and spun down (1300*g*, 3 minutes). The cell pellets were re-suspended in and washed with PBS. After a final spin down, keratinocyte media containing 1% gentamycin was used to re-suspend the cells and then plated in tissue culture dishes pre-coated with rat tail type I collagen (29 µg/ml, BD Bioscience). To isolate dermal fibroblasts, the dermis section was minced and placed in collagenase (1000 units/ml, Alfa Aesar, Ward Hill, MA) for 2 hours at 37°C. The tissue and cell mixture were passed through a cell strainer. Cells were spun down and washed with PBS. Cell pellets were plated with 20% fetal bovine serum (FBS)-containing DMEM medium. After the majority of cells have attached, the amount of FBS was reduced from 20% to 10%.

The isolation of pig keratinocytes follows the same procedures as above for human keratinocytes except the media is supplemented with a higher concentration of calcium (0.3 mM [Ca2+]) [39]. We found that pig keratinocytes were more sensitive to the human keratinocyte media and were unable to survive beyond the initial attachment of the cells. By testing varying concentrations of calcium, we found that 0.3 mM [Ca2+] provided the best support for pig keratinocyte growth. Pig dermal fibroblasts were able to survive and expanded for several passages using the same media as human dermal fibroblasts.

### Protein purification

cDNA cloning, production and purification of recombinant Hsp90α proteins have been carried out as previously described [7], [9].

### Cell migration assays

The colloidal gold migration assay was conducted as described previously [37]. Data from independent experiments (n≥3) were averaged and calculated (mean ± SD, *p*<0.05). In addition to rat tail collagen I, porcine collagen type I/III (45%/45%) from YO Proteins (Huddinge, Sweden) was also used in pig cell migration assays as a comparison.

### Statistic analyses

Data on animal wound healing were based on three or more independent experiments, multiple diabetic and control pigs. Data are presented as mean ± standard deviation (s.d.). Statistical significance for comparisons was determined by the Student's two-tailed t-test. A *p* value equal or less than 0.05 was considered statistically significant [40], [41].

### Ethics Statement

All animal studies were according to a porcine animal protocol approved by the University of Southern California's Institutional Animal Care and Use Committee (Protocol # 11581). Early termination, if necessary, of animals was in accordance with USDA's current space requirements found in the 8^th^ Edition of the Guide for the Care and Use of Laboratory Animals.

Human skin samples from patients were obtained under the protocol HS-11-00156 “Isolation of primary cells from various human tissues.” Samples were obtained during elective surgeries and are de-identified. These samples are to-be-discarded waste tissue from the operating room and therefore no formal consent is given. Patient information is not collected nor recorded. The protocol and consent procedures were approved by the University of Southern California Health Sciences Campus Institutional Review Board.
